#  Neutrophil to lymphocyte ratio as a predictor of treatment response and mortality in septic shock patients in the intensive care unit

**DOI:** 10.3906/sag-1901-105

**Published:** 2019-10-24

**Authors:** Rabia SARI, Zuhal KARAKURT, Mustafa AY, Muhammed Emin ÇELİK, Ülgen YALAZ TEKAN, Fulya ÇİYİLTEPE, Feyza KARGIN, Cüneyt SALTÜRK, Özlem YAZICIOĞLU MOÇİN, GÖKAY GÜNGÖR, Nalan ADIGÜZEL

**Affiliations:** 1 Department of Intensive Care, Hatay State Hospital, Hatay Turkey; 2 Department of Intensive Care, University of Health Sciences Süreyyapaşa Chest Diseases and Thoracic Surgery Research and Training Hospital, İstanbul Turkey; 3 Department of Intensive Care, Batman Regional Hospital, Batman Turkey; 4 Department of Intensive Care, Ahi Evran Thoracic and Cardiovascular Surgery Hospital, Trabzon Turkey; 5 Department of Neurology, Şişli Hamidiye Etfal Research and Training Hospital, İstanbul Turkey; 6 Department of Intensive Care, İstanbul Dr. Lütfi Kırdar Research and Training Hospital, İstanbul Turkey

**Keywords:** Septic shock, intensive care unit, respiratory failure, neutrophil to lymphocyte ratio

## Abstract

**Background/aim:**

While C-reactive protein (CRP) is a well-studied marker for predicting treatment response and mortality in sepsis, it was aimed to assess the efficacy of the neutrophil lymphocyte ratio (NLR) as a predictor of mortality and treatment response in sepsis patients in the intensive care unit (ICU).

**Materials and methods:**

In this retrospective cross-sectional study, sepsis patients were divided according to the presence of septic shock on the 1st day of ICU stay, and then subgrouped according to mortality. Patient demographics, acute physiologic and chronic health evaluation II and sequential organ failure assessment scores, NLR and CRP (on the 1st, 3rd, and last day in the ICU), microbiology data, antibiotic responses, ICU data, and mortality were recorded. Receiver operating characteristic (ROC) curves for the area under curve (AUC) were calculated for the inflammatory markers and ICU severity scores for mortality.

**Results:**

Of the 591 (65% male) enrolled patients, 111 (18.8%) were nonsurvivors with shock, 117 (19.8%) were survivors with shock, 330 (55.8%) were survivors without shock, and 33 (5.6%) were nonsurvivors without shock. On the 1st day of ICU stay, the NLR and CRP were similar in all of the groups. On the 3rd day of antibiotic response, the NLR was increased (11.8) in the nonresponsive patients when compared with the partially responsive (11.0) and responsive (8.5) patients. If the NLR was ≥15 on the 3rd day, the mortality odds ratio was 6.96 (CI: 1.4–34.1, P < 0.017). The NLR and CRP on the 1st, 3rd, and last day of ICU stay (0.52, 0.58, 0.78 and 0.56, 0.70, 0.78, respectively) showed a similar increasing trend for mortality.

**Conclusion:**

The NLR can predict mortality and antibiotic responsiveness in ICU patients with sepsis and septic shock. If the NLR is >15 on the 3rd day of postantibiotic initiation, the risk of mortality is high and treatment should be reviewed carefully.

## 1. Introduction

Early recognition of sepsis can lead to early treatment and a potential reduction in septic shock development [1]. Despite treatment improvements with the global sepsis campaign, patients with septic shock have a high mortality rate in the intensive care unit (ICU) [2–4].

Acute physiological and chronic health evaluation II (APACHE) and sequential organ failure assessment (SOFA) scores are well known mortality predictors in ICU patients with sepsis [5,6]. C-reactive protein (CRP) and procalcitonin are the most studied inflammatory markers of bacterial sepsis, particularly for making decisions regarding antibiotic treatment in the ICU [7–11]. SOFA and APACHE II scores are calculated to assess disease severity, treatment response, and risk of mortality in the ICU, and these are not easy to calculate at the bed side in daily practice.

There are some inflammatory markers, such as the neutrophil to lymphocyte ratio (NLR) and platelet to lymphocyte ratio (PLR), that are used to assess treatment response in sepsis patients for their simplicity [12,13]. 

Procalcitonin and CRP are the most used biomarkers to discriminate bacterial sepsis from other inflammatory diseases [14]. However, CRP can increase in both infectious and noninfectious diseases and, even though procalcitonin has a well-defined role as a sepsis biomarker, it has inconsistent and variable results for the diagnosis of infectious sepsis, not to mention it is expensive to assess [15,16]. Recently, studies have been undertaken on inflammatory markers obtained from complete blood counts (CBCs) with simple calculations for early recognition of infection and assessing treatment response [17–22]. Inflammatory markers obtained by CBCs, such as the NLR, PLR, platelet to mean platelet volume (MPV), have been assessed in different disease groups for their association with hospital mortality [12,13,22,23]. The NLR has been recommended as an infection biomarker since 2001 [24] and there have since been studies evaluating NLR alone, or as part of a multiple biomarker model, for the diagnosis of adult patients with suspected community onset sepsis [25,26]. High NLR combined with lymphocytopenia may be a better predictor of bacteremia than other routinely used parameters in the emergency department, such as CRP and the leucocyte count [26]. In addition, higher NLR and lymphocytopenia values were correlated with disease severity [24].

It was aimed herein to determine whether NLR obtained from CBCs and with simple calculation can be used to predict mortality and treatment response in patients with sepsis and septic shock in the ICU.

## 2. Materials and methods

This study was performed in a level 3 ICU, in a chest diseases and thoracic surgery training and research hospital after approval from the hospital’s local ethics committee, as prescribed by the Helsinki Declaration. Due to the retrospective nature of the study, no informed consent was obtained from the patients.

The ICU was operated by the same intensivist/pulmonologist team over a 7-day period and each ICU team followed the same ICU procedures, as defined by written protocols (such as sepsis, mechanical ventilation, weaning, etc.).

### 2.1. Patients

**Figure 1:**
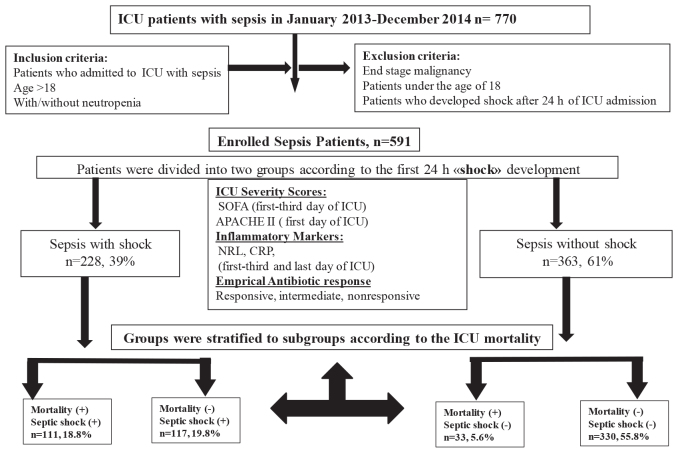
Patient flow chart.

This was designed as a retrospective, observational, and cross-sectional study, from January 2013 to April 2015. Patient data, including inclusion and exclusion criteria, the presence of septic shock on admission to the ICU, ICU mortality, and assignment according to the presence of septic shock, are summarized in a flow chart in Figure 1. Patients admitted to the ICU had pulmonary sepsis and septic shock that was either community acquired pneumonia or acute exacerbation of chronic obstructive pulmonary disease. Patients were stratified according to ICU mortality; categorized as early death (day 1 to 4), or late death (day 5 or after) [27].

**Table 1:** A comparison of sepsis patient demographics, ICU data, and inflammatory markers relative to the presence of septic shock.

	Septic shock on admission to the intensive care unit				
	Absent, n = 392		Present, n = 199		
Variable	n	Median (IQR)	n	Median (IQR)	P-value
Age	392	65 (56–75)	199	71 (62–79)	<0.001
Sex, Male n (%)	363	248 (63)	228	133 (67)	0.39
BMI, kg/m2	361	23 (21–28)	183	23 (20–28)	0.36
Comorbid diseases					
Diabetes mellitus	91	23%	51	26%	0.51
Chronic obstructive pulmonary disease	196	50%	95	48%	0.58
Hypertension	158	40%	84	42%	0.67
Coronary arterial disease	49	13%	31	16%	0.30
Atrial fibrillation	38	10%	28	14%	0.11
Cerebrovascular disease	27	7%	20	10%	0.17
Lung cancer	37	9%	29	15%	0.06
Extra-pulmonary cancer	21	5%	14	7%	0.41
Chronic kidney disease	12	3%	9	5%	0.36
Obesity hypoventilation syndrome	24	6%	7	4%	0.17
ICU data					
APACHE II score on admission	388	18 (15–23)	197	29 (23–33)	0.001
SOFA score on admission	361	3 (2–4)	228	7 (5–9)	0.001
SOFA score on the 3rd day of ICU stay	325	2 (2–3)	202	5 (3–8)	0.001
Invasive mechanical ventilation,	94	26%	42	18%	0.001
Noninvasive mechanical ventilation,	257	71%	130	57%	0.001
Noninvasive ventilation failure,	32	9%	54	24%	0.001
Length of ICU stay, days	392	6 (4–9)	199	7 (4–12)	0.14
Invasive mechanical ventilation duration, days	120	3 (2–6)	160	4 (2–8)	0.02
Antibiotic responsiveness					
Responsive	211	58%	69	30%	0.001
Intermediate	130	36%	123	54%	
Nonresponsive	22	6%	36	16%	
CRP, mg/L					
1st day of ICU stay	292	90 (33–160)	174	98 (44–182)	0.13
3rd day of ICU stay	263	73 (2135)	167	125 (62–182)	0.001
Last day of ICU stay	304	43 (20–198)	182	82 (42–155)	0.001
NLR					
1st day of ICU stay	363	10.50 (5.76–19.56)	228	13.48 (7.54–23.48)	0.001
3rd day of ICU stay	326	8.64 (5.23–15.33)	199	10.97 (6.73–18.41)	0.002
Last day of ICU stay	356	5.93 (3.93–10.25)	220	8.19 (4.47–16.39)	0.001

IQR: interquartile range. APACHE II: acute physiologic and chronic health evaluation II. SOFA: sequential organ failure assessment, NLR: neutrophil to lymphocyte ratio, CRP: C-reactive protein.

Study endpoint: ICU mortality and discharge from ICU in patients with septic shock and sepsis alone.

### 2.2. Definitions

#### 2.2.1. Systemic inflammatory response syndrome (SIRS) and sepsis-septic shock 

Definitions used in the Surviving Sepsis Campaign; an International Guideline for the Management of Severe Sepsis and Septic Shock (2012), were deemed valid for the study period. SIRS is defined by the following criteria: a pulse rate of 90/min and above or 2 standard deviations above the normal value for age; a respiratory rate of 20 and above or a partial carbon dioxide pressure (PaCO2) of 32 mmHg and below; alteration of consciousness; a white blood cell (WBC) count greater than 12,000 µL–1 or below 4000 µL–1; a temperature less than 36 °C or greater than 38.3 °C; plasma glucose levels of >40 mg/dL; a plasma CRP level 2 standard deviations above the normal value; and an inspired oxygen fraction of the partial arterial oxygen concentration (PaO2/FiO2) of <300.

Patients with 2 or more SIRS criteria and with a suspected or proven infection were defined as having sepsis [28]. Sepsis induced hypotension was defined as a systolic arterial pressure of £90 mmHg, a mean arterial pressure of £65 mmHg, or a decrease in systolic arterial pressure of >40 mmHg in patients with no other health issues causing hypotension. As a vasopressor, noradrenalin was initiated at 0.01–3 µg/kg/min intravenous infusion, and if necessary, dopamine was added at 5–20 µg/kg/min.

### 2.3. Recording data

Patient demographics (age, sex, body mass index (BMI) [kg/m2]), arterial blood gas (ABG) values (Rapidlab, Bayer, Leverkusen, Germany), and the presence of comorbidities, such as chronic obstructive pulmonary disease (COPD) [29], hypertension [30], diabetes mellitus [31], lung cancer, coronary artery disease, atrial fibrillation, arrhythmia, Alzheimer’s disease, chronic renal failure, and obesity hypoventilation syndrome, were recorded on admission and discharge from the ICU. CBC values, including the WBC, hematocrit (Htc), platelet, MPV, neutrophil count, lymphocyte count on the 1st, 3rd, and last day of ICU stay, calculated NLR, and CRP on the 1st, 3rd, and last day of ICU stay, were also recorded. In addition, biochemical values on ICU admission and discharge were recorded. The APACHE II score was recorded on the day of ICU admission, and the SOFA score was recorded on the day of admission and 3rd day in the ICU. The use of invasive mechanical ventilation (IMV) and noninvasive mechanical ventilation (NIMV) was noted. Culture results, duration of ICU stay in days, and ICU mortality were also recorded.

### 2.4. Calculations

 The NLR was calculated as the neutrophil absolute value over the lymphocyte absolute value. According to the literature, the patients were grouped according to whether their NLR values were above or below 10 and 15 on the 1st, 3rd, and last day of ICU stay [32].

### 2.5. Mechanical ventilation application

In the ICU, all patients not absolutely contraindicated were initially given NIMV. Contraindications included: 1) heart and/or pulmonary arrest, 2) unconsciousness (excluding hypercarbia), 3) nonrespiratory organ failure, severe encephalopathy, shock, heart pathology leading to unstable hemodynamics, or severe upper gastrointestinal tract bleeding, 4) lack of airway protection, 5) inability to remove secretions, 6) risk of aspiration, 7) upper airway obstruction, and 8) facial surgery, trauma, deformity, or burning [33]. NIMV failure was defined as the continuation of acidosis in ABG after NIMV application, aggravation of respiratory distress, mask and NIMV incompatibility, hemodynamic instability, cardiac arrest, respiratory failure, and a loss of consciousness [34]. Patients with NIMV failure, or in cases where NIMV application was contraindicated, received IMV via endotracheal intubation. IMV was defined as volume- or pressure-controlled, assisted control ventilation, performed based on the 6–8 mL/kg tidal volume for ideal body weight, or a plateau pressure equal to or less than 30 cmH2O. For COPD patients, FiO2 was titrated to 88%–92% oxygen saturation, and for cardiac ischemic patients, it was titrated to 95% and above. Positive end expiratory pressure (PEEP) was titrated, taking into account hemodynamic parameters in patients with COPD, to 5–6 cmH2O, together with the mean arterial pressure (65 mmHg and above) and plateau pressure (30 cmH2O and below). Patients undergoing IMV were followed up with a fentanyl/midazolam/propofol infusion as sedation and pain palliation was assessed using the Richmond agitation and sedation scale [35]. Sedation was interrupted daily and the patients’ clinical condition was assessed. 

### 2.6. Weaning protocol

For those patients with improved clinical and laboratory values, mechanical ventilation support was reduced and a spontaneous breathing trial was performed. The spontaneous breathing trial was performed once per day using a 30-min T-tube test or pressure support ventilation with a PEEP of 5 cmH20 and pressure support of 8 cmH2O [36].

### 2.7. Microbiology 

Deep tracheal aspirations were cultured for intubated patients. Sputum cultures were obtained for nonintubated patients. In patients with a temperature of <36 °C or >38 °C, blood cultures were taken for aerobes and anaerobes. In the study period, blood cultures were not taken from patients with normothermia, as per the hospital infectious committee protocol for ICU sepsis. 

### 2.8. Antibiotic response 

· Responsive: A sensitive culture response or significant improvement in the clinical and laboratory values to treatment showed no culture or a nonreproductive culture response.

· Partially responsive: Treatment with a moderately sensitive culture response or partial improvement in the clinical and laboratory values, in that patients had a noncultured or nonreproductive culture response.

· Nonresponsive: Treatment with a resistant culture response or significant deterioration in the clinical and laboratory values, in that patients had a noncultured or nonreproductive culture response.

### 2.9. Statistical analysis

Study data were analyzed using the SPSS v.20.0 (IBM Corp., Armonk, NY, USA) portable package program. The continuous numerical values of the binary groups were compared using Student’s t-test for uniform distribution and expressed as the mean ± standard deviation. Nonuniform distribution was assessed using the Mann–Whitney U test and median quarter-to-quarter ratio. Dichotomous values were summarized using the chi-square test. Comparisons of 2 or more groups were compared using the Kruskal–Wallis test for unevenly distributed data. Assessment of mortality markers was shown using the receiver operating characteristic (ROC) curve and the area under the curve (AUC). Multilogistic regression analysis was performed using known mortality markers affecting mortality in the ICU (APACHE II and SOFA scores), inflammatory markers (CRP, NLR), demographic characteristics affecting mortality, such as age, male sex, BMI, and comorbidities found to be significant in binary variants (heart failure, IMV, antibiotic sensitivity response, shock upon ICU admission, and length of ICU stay). P < 0.05 was considered statistically significant.

## 3. Results

Distribution of the study patients into the defined groups is summarized in Figure 1. During the study period, 591 of 770 patients were included in the study (381 [65%] were male). The median age was 67 (19–99) years old. Septic shock within the first 24 h was seen in 39%, and ICU mortality occurred in 144 (24.4%) of the 591 patients.

The demographic characteristics of the patients relative to the presence of septic shock are shown in Table 1. Patients with septic shock were found to have a significantly higher APACHE II score on ICU admission; they were older and had more cardiac arrhythmias than those without septic shock (Table 1). The inflammatory markers and antibiotic responses are summarized in Table 1. 

Patient NLR and CRP values relative to antibiotic responsiveness over the duration of the ICU stay are summarized in Table 2. The NLR and CRP values on the 3rd day in the ICU were significantly higher in patients with partial resistance and resistance to antibiotics, compared to the antibiotic-sensitive group. Following treatment, CRP values were similar between the groups on the last day of ICU stay, but the NLR was statistically lower in the antibiotic-sensitive group (Table 2). 

**Table 2:** Comparison of the NLR and CRP values in response to antibiotic therapy in septic shock patients in the ICU.

	Responsive to antibiotherapy		Partially responsive to antibiotherapy		Nonresponsive to antibiotherapy		
Inflammatory markers	n = 280	Values, median (IQR)	n = 253	Values, median (IQR)	n = 58	values, median (IQR)	P-value
CRP, mg/L							
1st day of ICU stay	213	99 (38–175)	204	90 (36–157)	49	95 (43–182)	0.86
3rd day of ICU stay	201	79 (27–140)	190	105 (42–167)	39	115 (84–180)	0.005
Last day of ICU stayv	245	51 (22–108)	199	62 (24–139)	42	74 (20–121)	0.35
NLR							
1st day of ICU stay	280	11.2 (6.6–17.9)	253	12.7 (6.2–25.1)	58	10.0 (5.4–20.7)	0.17
3rd day of ICU stay	256	8.5 (5.5–14.1)	220	11.0 (6.2–18.0)	49	11.8 (6.5–21.7)	0.008
Last day of ICU stay	273	5.7 (3.8–9.0)	247	9.7 (4.5–15.9)	56	8.4 (4.2–15.7)	0.001

IQR: interquartile ratio, nonparametric Kruskal–Wallis test.

Table 3 summarizes the ICU data and inflammatory markers of those patients with pulmonary sepsis relative to mortality in the ICU within 5 days. Age and sex were similar in both the survivors and nonsurvivors within 5 days. Apart from lung cancer and COPD, comorbid diseases were similar whether the patients died within 5 days or not. A significantly higher rate of nonresponsiveness to antibiotic treatment, IMV application, and the presence of septic shock were found in those patients who died within 5 days. Among the inflammatory markers, only the NLR was significantly higher on the 1st day of ICU stay in those patients who died within 5 days. 

**Table 3:** ICU data and inflammatory markers for patients with pulmonary sepsis relative to mortality in the ICU within 5 days.

	Survivors in 5 days		Nonsurvivors in 5 days		P-value
	n	Values	n	Values	
Age	535	67 (57–77)	56	71 (57–80)	0.19
Sex, Male	347	65%	34	61%	0.54
Body mass index kg/m2	495	23 (21–28)	22	22 (19–25)	0.017
Comorbid diseases					
Diabetes mellitus	130	24%	12	21%	0.63
Hypertension	220	41%	22	39%	0.78
COPD	275	52%	16	29%	0.001
Coronary heart diseases	75	14%	5	9%	0.29
Lung cancer	50	9%	16	29%	0.001
Arrhythmia	135	25%	15	27%	0.80
APACHE II score, 1st day of ICU stay	535	20 (16–27)	55	30 (25–38)	0.001
SOFA score, 1st day of ICU stay	535	4 (2–6)	55	8 (5–10)	0.001
SOFA score, 3rd day of ICU stay	503	3 (2–5)	24	9 (6–10)	0.001
Septic shock	184	34%	44	79%	0.001
Culture study	363	68%	28	50%	0.007
Positive culture	91	25%	12	43%	0.039
Antibiotic responsiveness					
Responsive	273	51%	7	13%	0.001
Intermediate	221	41%	32	57%	
Nonresponsive	41	8%	17	30%	
Mechanical ventilation					
Noninvasive	363	68%	24	43%	0.001
Noninvasive failure	76	14%	10	18%	0.46
Invasive	243	45%	37	66%	0.003
CRP, mg/L					
1st day of ICU stay	427	94 (34–165)	39	91 (50–187)	0.48
3rd day of ICU stay	419	94 (36–153)	11	180 (116–288)	0.006
Last day of ICU stay	464	53 (21–111)	22	193 (118–333)	0.001
NLR					
1st day of ICU stay	535	11.23 (6.19–-20.57)	56	17.47 (9.28–27.12)	0.016
3rd day of ICU stay	501	9.33 (5.67–15.67)	24	17.40 (8.42–33.80)	0.004
Last day of ICU stay	529	6.24 (3.95–11.01)	47	17.74 (9.58–31.97)	0.001

Table 4 shows a comparison of the pulmonary sepsis patients; demographics, ICU data, and inflammatory markers of survivors versus nonsurvivors. Older age, a higher level of inflammatory markers, and a higher rate of nonresponsiveness to antibiotherapy were found in the nonsurvivors when compared with the survivors.

**Table 4:** Comparison of pulmonary sepsis patients; demographics, ICU data and inflammatory markers of survivors versus nonsurvivors.

	Survivors	Nonsurvivors	P-values
Number of patients	447	144	-
Male, %	64.4	64.6	0.97
Age, years	66 (57–75)	74 (60–81)	0.001
Body mass index kg/m2	23 (21–28)	23 (20–26)	0.07
Comorbid diseases, n (%)			
Hypertension	194 (43.5)	48 (33.3)	0.031
Coronary artery diseases	66 (14.8)	14 (9.7)	0.12
Diabetes mellitus	110 (24.6)	32 (22.2)	0.56
Arrhythmias	102 (22.8)	48 (33.3)	0.012
Chronic obstructive pulmonary diseases	240 (53.8)	51 (35.4)	0.001
Cerebrovascular accident	416 (93.1)	128 (88.9)	0.11
Obesity hypoventilation	30 (6.7)	1 (0.7)	0.005
Chronic renal failure	12 (2.7)	9 (6.7)	0.044
Lung cancer	34 (7.6)	32 (22.2)	0.001
ICU data			
APACHE II on admission to the ICU	19 (16–24)	29 (24–35)	0.001
SOFA score on admission to the ICU	3 (2–5)	7 (5–10)	0.001
SOFA score on the 3rd day of ICU stay	3 (2–4)	6 (4–9)	0.001
ICU length of stay, days	6 (4–9)	5 (3–12)	0.23
Septic shock	117 (26.2)	111 (77.1)	0.001
Noninvasive ventilation, n (%)	304 (68)	83 (57.6)	0.023
Noninvasive ventilation days	4 (2-7)	2 (1-4)	0.001
Noninvasive failure to invasive ventilation, n (%)	45 (10.1)	41 (28.5)	0.001
Invasive ventilation, n (%)	173 (38.7)	107 (74.3)	0.001
Invasive ventilation days	3 (2-6)	7 (5-10)	0.008
Sepsis protocol			
Empirical antibiotic, n (%)			
· responsive	262 (58.6)	18 (12.5)	0.001
· intermediate	164 (36.7)	89 (61.8)	
· unresponsive	21 (4.7)	37 (25.7)	
Insulin infusion, n (%)	22 (4.9)	19 (13.2)	0.001
Steroid (60 mg/day), n (%)	10 (2.2)	12 (8.3)	0.001
Culture sample, N (%)	294 (65.8)	97 (67.4)	0.73
Positive culture sample, n (%)	62 (21.1)	41 (41.8)	0.001
Inflammatory markers			
CRP, mg/L			
1st day of ICU stay	89 (29–163)	112 (56–169)	0.029
3rd day of ICU stay	81 (28–145)	126 (91–194)	0.001
Last day of ICU stay	44 (20–92)	136 (81–219)	0.001
NLR			
1st day of ICU stay	10.36 (5.94–19.56)	14.85 (7.54–26.58)	0.006
3rd day of ICU stay	8.56 (5.25–15.27)	13.02 (8.40–19.90)	0.001
Last day of ICU stay	5.50 (3.69–8.71)	15.74 (8.00–28.33)	0.001

Table 5 shows a comparison of the patient APACHE II and SOFA scores, NLR, and CRP in patients with or without the occurrence of septic shock and mortality. APACHE II scores on ICU admission were significantly higher in those patients who died in the ICU, independent of septic shock. APACHE II values were similar between those patients who were in septic shock and those that died in the ICU. Patients without septic shock had similar SOFA scores on ICU admission, regardless of their mortality in the follow-up period. There was a significant difference between all of the other patient groups. The NLR on ICU admission was significantly higher in those patients with septic shock and those who died in the ICU, when compared with patients without septic shock and survivors. On the 3rd day in the ICU, the SOFA scores were statistically different in the 4 subgroups. In the subgroup of survivors with no septic shock, the CRP values on the 3rd day in the ICU were significantly lower than in the other groups, and their NLR values were significantly lower than in the septic shock patients, independent of mortality. The CRP values of the survivors on the last day of ICU stay were statistically similar, regardless of the presence of septic shock, but these values were significantly different in the nonsurvivor subgroups. The NLR values on the last day of ICU stay were significantly higher in the nonsurvivors, independent of the presence of septic shock (Table 5).

**Table 5:** Comparison of the inflammatory markers and ICU severity scores with the presence of mortality and septic shock in pulmonary sepsis patients in the ICU.

Variables	aShock – mortality –		bShock – mortality +		cShock + mortality -		dShock + mortality +		P-values
	n = 330	Values	n = 33	Values	n = 117	Values	n = 111	Values	
ICU admission									
APACHE II score	327	17 (15–22)	32	24 (20–27)	117	25 (20–30)	109	30 (26–36)	a vs. b < 0.001a vs. c < 0.001a vs. d < 0.001b vs. c = 0.87b vs. d < 0.001c vs. d < 0.001
SOFA score	329	3 (2–4)	32	3 (3–4)	117	6 (5–8)	111	8 (6–10)	a vs. b = 0.68a vs. c < 0.001a vs. d < 0.001b vs. c < 0.001b vs. d < 0.001c vs. d < 0.001
CRP	264	86 (3160)	28	113 (52–152)	95	91 (27–189)	79	102 (57–176)	a vs. b = 0.99a vs. c = 0.97a vs. d = 0.24b vs. c = 0.99b vs. d = 0.24c vs. d = 0.61
NLR	330	10.1(5.7–18.9)	33	13.6 (8.1–22.2)	117	12.7 (7.9–21.5)	111	15.7 (7.5–26.6)	a vs. b = 0.86a vs. c = 0.58a vs. d = 0.009b vs. c = 0.99b vs. d = 0.73c vs. d = 0.38
3rd day of ICU stay									
SOFA score	298	2 (2–3)	27	4 (3–5)	117	4 (3–6)	85	8 (5–10)	a vs. b = 0.000a vs. c = 0.000a vs. d = 0.000b vs. c = 0.91b vs. d = 0.000c vs. d = 0.000
CRP	242	70 (24–132)	21	112 (80–180)	100	119 (54–175)	67	135 (97–196)	a vs. b = 0.045a vs. c = 0.001a vs. d = 0.000b vs. c = 0.94b vs. d = 0.78c vs. d = 0.09
NLR	298	8.4 (5.0–15.1)	28	12.9 (9.2–17.9)	115	10.1 (6.2–15.4)	84	13.5 (8.3–20.8)	a vs. b = 0.05a vs. c = 0.045a vs. d = 0.000b vs. c = 0.75b vs. d = 0.99c vs. d = 0.32
Last day of ICU stay									
CRP	282	40 (18–92)	22	103 (51–202)	107	53 (23–92)	75	151 (85–221)	a vs. b < 0.001a vs. c = 0.61a vs. d < 0.001b vs. c < 0.001b vs. d = 0.97c vs. d < 0.001
NLR	324	5.5 (3.8–8.6)	32	15.1 (8.2–24.5)	117	5.4 (2.4–8.7)	103	16.0 (8.0–30.5)	a vs. b < 0.001a vs. c = 0.99a vs. d <0.001b vs. c < 0.001b vs. d = 0.31c vs. d < 0.001

*Non-parametric Kruskal Wallis Test, median values (25%–75%).

Potential mortality risk factors assessed in the pulmonary sepsis patients during their ICU stay using binary logistic regression are summarized in Table 6. NLR values of 10 and 15, CRP of 100 mg/L and above on admission to the ICU, age and BMI, arrhythmia, shock on admission, number of days in the ICU, and IMV application were not found to be risk factors for mortality in the patient group. However, mortality was shown to be increased by 35.4 times in patients with resistance to the empirical antibiotic treatment (Table 6), and 10.3-fold in patients with partial antibiotic resistance. Mortality increased 7-fold in patients with NLR values of 15 and above on the 3rd day of ICU stay. Each value increase in the APACHE II score on admission to the ICU increased mortality 1.13-fold. Each value increase in the SOFA score on the 3rd day of ICU stay increased mortality 1.5-fold, while each value increase in the SOFA score on the 1st day of ICU stay decreased mortality 0.3-fold (Table 6).

**Table 6:** Mortality risk factors evaluated by binary logistic regression in pulmonary sepsis patients in the ICU.

	Odds ratio	CI 95% lower-upper	P-values
Antibiotic response			
Partially responsive	10.3	3.3–31.5	<0.001
Nonresponsive	35.4	7.9–158.3	<0.001
3rd day NLR of ≥15	6.96	1.42–34.1	0.017
3rd day NLR of ≥10	2.71	0.63–11.63	0.18
1st day NLR of ≥15	1.27	0.32–4.92	0.72
1st day NLR of ≥10	0.53	0.14–2.01	0.36
3rd day SOFA score	1.57	1.23–2.01	<0.001
1st day SOFA score	0.69	0.54–0.89	0.005
1st day APACHE II score	1.12	1.03–1.23	0.005
1st day CRP of >100	1.04	0.40–2.68	0.92
3rd day CRP of >100	0.64	0.21–1.97	0.44
Age	0.98	0.94–1.02	0.36
BMI	0.96	0.89–1.03	0.27
IMV	2.38	0.73–7.76	0.15
Arrhythmia	2.48	0.85–7.17	0.09
Shock on admission	2.54	0.92–6.99	0.07
ICU days	1.00	0.94–1.06	0.85

CI: confidence interval, BMI: body mass index, IMV: invasive mechanical ventilation.

**Figure 2:**
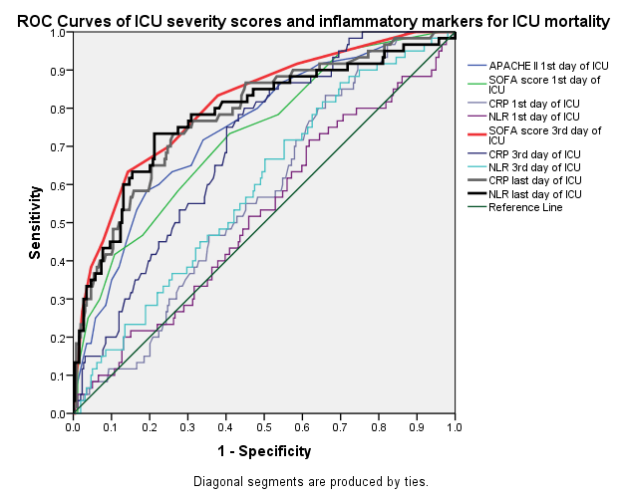
Mortality prediction markers in pulmonary sepsis patients in the ICU; ROC curve analysis.

A ROC analysis to predict ICU mortality based on the APACHE II scores (on ICU admission), SOFA scores (on ICU admission and on the 3rd day of ICU stay), and NLR and CRP values (1st, 3rd and last day of ICU stay) is shown in Figure 2, and the AUC values are summarized in Table 7. The AUC values were clinically and statistically significant for the APACHE II and SOFA scores on admission to the ICU, the SOFA scores and CRP values on the 3rd day of ICU stay, and CRP and NLR values on the last day of ICU stay (Table 7).

**Table 7:** Area under the curve for mortality in pulmonary sepsis patients in the ICU.

Test result variable(s)	Area	P-values	Asymptotic 95% confidence interval	
			Lower bound	Upper bound
SOFA, 3rd day of ICU stay	0.807	0.001	0.744	0.870
CRP, last day of ICU stay	0.780	0.001	0.712	0.849
NLR, last day of ICU stay	0.778	0.001	0.705	0.851
APACHE II, 1st day of ICU stay	0.740	0.001	0.672	0.809
SOFA, 1st day of ICU stay	0.723	0.001	0.652	0.795
CRP, 3rd day of ICU stay	0.699	0.001	0.632	0.766
NLR, 3rd day of ICU stay	0.579	0.06	0.501	0.658
CRP, 1st day of ICU stay	0.562	0.14	0.487	0.638
NLR, 1st day of ICU stay	0.515	0.73	0.431	0.599

## 4. Discussion

This study showed that CRP was similar in patients with sepsis and septic shock that developed within the first 24 h following admission to the ICU. Significantly higher NLR levels were found on ICU admission in patients with septic shock when compared with those with sepsis alone. The NLR and CRP values behaved similarly relative to the patient severity and response to treatment during the ICU stay and ICU outcome. The CRP and NLR values on the last day of ICU stay were significant predictors of ICU mortality. The present study also showed that if treatment with inappropriate antibiotics was initiated, mortality increased 35-fold. NLR values equal to or greater than 15 on the 3rd day of ICU stay were associated with a 7-fold increase in mortality. The NLR and CRP showed similar changes when monitoring the response to antibiotics.

### 4.1. ICU mortality predictors: APACHE II and SOFA scores, NLR, and CRP

Recently, Yu et al. compared different evaluating systems to predict the prognosis of patients with infections outside of the ICU. During the first 12 h before clinical worsening, they reported the AUC for the SOFA score as 0.78, and 0.72 for the APACHE II score [37]. A very recent study by Jain at al., that investigated the SOFA score/ICU mortality relationship, similarly determined that the higher the SOFA score on the 1st, 3rd, and 5th day of ICU stay, the higher the ICU mortality. However, they also showed that SOFA scores on the 7th and 9th day were not associated with mortality [38]. In the present study, the AUC for SOFA score on the 3rd day was the highest value among the other severity indices (Figure 2 and Table 7). 

Salciccioli et al. assessed NLR values as a risk factor for mortality in the general ICU. They separated patients into 4 groups based on their NLR values and reported that the presence of sepsis was not an additional risk for mortality when compared with nonsepsis patients [39]. In the same study, 3rd and 4th quarter NLR values were found to be a risk factor for mortality in general ICU patients. The mortality risk coefficients for the 3rd quarter (75%, NLR = 8.90–16.21) and 4th quarter (75%–100%, NLR of ≥16.21) NLR were 1.35 and 1.75, respectively. These were similar to the NLR values of the nonsurvivor sepsis patients in the ICU in the current study (Table 4). Riché et al. investigated the relationship between NLR in patients with septic shock and time of death, i.e. early death (before 5 days) and late death (5th day and after) during ICU stay [27]. The patient groups had either abdominal sepsis (n = 130) or extra-abdominal sepsis (n = 31), and their NLR median values on admission to the ICU were similar (9.0 [interquartile range (IQR) of 4.6–17.9] and 11.5 [IQR of 5.5–18.5], respectively) [27]. In contrast, in the present study, the septic patient populations were of a pulmonary sepsis origin and the early death patients had significantly higher NLR values than in the study by Riché et al. (median NLR 17 vs. 5) (Table 5). It was also shown that early nonsurvivor sepsis patients had a significantly higher rate of septic shock, and higher inflammatory marker values and ICU severity scores than sepsis patients without shock.

### 4.2. Antibiotic response: CRP, NLR, and procalcitonin

Schmit et al. investigated the clinical course of CRP in response to initial antibiotic therapy in sepsis patients. They found CRP to predominantly increase within the first 48 h of treatment, and by at least 2.2 mg/dL [40]. These findings were similar in patients in the current study who were partially sensitive or resistant to antibiotics; they tended to have elevated CRP levels on the 3rd day of ICU stay. Renny et al. indicated that a drop in CRP of 50 mg/dL or more from ICU admission to the 4th day of ICU stay was a good cutoff value for anticipating recovery in sepsis patients [41]. The CRP and NLR values on the 1st and 3rd day of ICU stay in sepsis patients in the present study were assessed according to guidelines that suggested that the response to the initial antibiotic therapy should be assessed by a regression in biomarkers and clinical response within the first 48–72 h [42]. While the CRP and NLR values increased on the 3rd day in the partially-sensitive and antibiotic-resistant patient groups, the CRP and NLR values decreased on the last day following treatment modification. 

Gürol et al. assessed procalcitonin as a reference and predictor of sepsis and septic shock and compared it with the NLR, CRP and leucocyte counts using ROC analysis [43]. They concluded that the strongest indicator for sepsis was NLR. If the NLR was equal to 5 or above, it indicated a sepsis diagnosis, and the requirement for treatment and follow-up of infection in a critically ill patient. The authors defined procalcitonin values from 2–10 ng/mL in patients with sepsis and 10 ng/mL in patients with septic shock. They also defined values for NLR in the range of 13–15 for patients with sepsis and over 15 for patients with septic shock. Unlike their study, in which admission values with suspected bacteremia were taken into account, patients in the present study had pulmonary sepsis. Significantly higher values of NLR and CRP were observed in patients with sepsis and septic shock in the ICU (Table 1). After empirical antibiotic therapy, the NLR and CRP were significantly higher on the 3rd day in the nonresponsive sepsis/septic shock patients (Table 2).

### 4.3. Limitations

A limitation of this study was that it was retrospective and single-centered. However, given that the center where the patients’ records were taken from and the study was done at had a consistent team of ICU physicians who implemented a specific sepsis protocol, we can say that the data were controllable, even if acquired retrospectively. We believe that the results presented herein are significant, as the number of patients assessed was high and they were treated and followed by a specific group of physicians. A further limitation was that the sepsis patients were of a pulmonary origin and the study was conducted in the pulmonary ICU. Thus, we could not generalize our results for other causes of sepsis. Nevertheless, pulmonary originated sepsis is a frequently occurring type of sepsis encountered by intensivists in the intensive care [44]. We believe that our data is valuable for ICU physicians in clinical practice.

### 4.4. Conclusions

For patients with sepsis and septic shock, NLR and CRP values on admission to the ICU (within the first 24 h) are not as valuable as the 1st day SOFA and APACHE II scores for predicting mortality. The NLR is as valuable as CRP for assessing the response to empirically initiated antibiotic treatment in sepsis patients. NLR values of >15 on the 3rd day of ICU stay can be used to predict mortality. We suggest using NLR values for sepsis patients in the first 3 days to assess follow-up and antibiotic treatment. Further studies are required to clarify these results and to assess the utility of NLR as a predictor of mortality and treatment response in other sepsis and septic shock patient populations.
